# Exploring the impact of online and offline teaching methods on the cognitive abilities of medical students: a comparative study

**DOI:** 10.1186/s12909-023-04549-x

**Published:** 2023-08-08

**Authors:** Yangting Xu, Lu Wang, Peidi Li, Hong Xu, Ziqi Liu, Ming Ji, Ziqiang Luo

**Affiliations:** 1 Xiangya School of Medicine, Central South University, Changsha, China; 2 Shanghai Key Laboratory of Psychotic Disorders, Brain Health Institute, Shanghai Mental Health Center, National Center for Mental Disorders, Shanghai Jiaotong University School of Medicine, Shanghai, China; 3 Xiangya School of Pharmacy, Central South University, Changsha, China; 4 Department of Physiology, School of Basic Medicine, Central South University, Changsha, China

**Keywords:** Online teaching, Offline teaching, Learning Effect, Medical Education, Cognitive skills

## Abstract

**Background:**

Online education has become increasingly popular, but research on the effectiveness of different teaching models in developing cognitive skills is limited. This study investigated the relationship between different teaching models (online and offline) and the development of cognitive skills in clinical medicine students.

**Methods:**

Survey data were collected from 2018 entry students who participated in online teaching and 2019 entry students in offline teaching at Xiangya School of Medicine, Central South University. National Quality Open Courses (NQROC) were provided to both groups of students. The study examined the total score of physiology final exam, score of each type of question, and NQROC learning engagement in different score segments under the two teaching models. Non-parametric statistical methods were utilized to analyze the total score of physiology final exam, score of each type of question, and the NQROC learning engagement. Spearman’s rank correlation was utilized to analyze the relationship between the score of physiology final exam and the students’ NQROC learning engagement.

**Results:**

The study found no statistically significant difference in the total score, short-answer questions (SAQs) score, and case study questions (CSQs) score between online and offline teaching models. However, the multiple-choice questions (MCQs) score was higher in the online teaching model (*Z*=-4.249, *P* < 0.001), suggesting that online teaching may be an effective way to improve lower-order cognitive skills among students. In contrast, low-achieving students had higher total scores (*Z*=-3.223, *P* = 0.001) and scores in both MCQs (*Z*=-6.263, *P* < 0.001) and CSQs (*Z*=-6.877, *P* < 0.001) in the online teaching model. High-achieving students in the online teaching model had higher total scores (*Z*=-3.001, *P* = 0.003) and MCQs scores (*Z*=-5.706, *P* < 0.001) but lower scores in CSQs (*Z*=-2.775, *P* = 0.006). Furthermore, students’ NQROC learning engagement was greater in the online teaching model.

**Conclusions:**

The results of this study suggested that online teaching was not statistically significantly different from offline in cognitive domains and was more desirable than offline in strengthening lower-order cognitive skills. However, it was important to note that offline teaching may be more effective in reinforcing higher-order cognitive skills among high-achieving students. In conclusion, this study provided important insights into the effectiveness of different teaching models in developing cognitive skills among medical students and highlighted the potential benefits of online teaching in enhancing students’ lower-order cognitive skills.

**Supplementary Information:**

The online version contains supplementary material available at 10.1186/s12909-023-04549-x.

## Background

With the rapid advancement of the Internet in the 21st century, online teaching models are gaining increasing popularity in medical education [1]. Online education has revolutionized the landscape of modern education by providing learners with enhanced flexibility and diverse options for content delivery, unconstrained by geographical boundaries and time limitations [2]. In the realm of medical education, the benefits of online learning have been harnessed to introduce innovative pedagogical practices into the curriculum. Extensive global research has been conducted to compare online and face-to-face learning in medical education, including investigations into student preferences for different teaching models [3] and the correlation between academic performance, course difficulty, and perceived effectiveness [1]. While some studies have postulated that online instruction is as effective as traditional teaching based on student feedback [4], they have also demonstrated similar learning outcomes, satisfaction, and comprehension for both online and offline instruction [5]. However, other studies have indicated that purely online instruction does not show more promise than traditional methods [6]. A mixed methods study reveals that teachers and students rate face-to-face learning more favorably than online learning [7]. Another survey reveals that both types of learning are equally effective in terms of perceived ability to increase knowledge, but online learning is considered less effective than face-to-face learning in terms of enhancing social skills and overall improvement [8]. Although there have been some initial findings about the effectiveness of online teaching in terms of student feedback, academic performance, learning engagement, understanding level and social skills, knowledge about its impact on the cognitive development of medical students remains limited.

Cognition is one of the three key skills for learning and education plays a vital role in advancing individuals’ cognitive abilities [9]. Proficient teachers are adept at assessing their students’ cognitive states during classroom instruction and adapt the learning experience in real-time to promote comprehension and positive attitudes toward education [10]. Incorporating Bloom’s taxonomy into learning can assist educators in constructing and generating ideas to enhance the effectiveness of their classes [11]. Bloom’s taxonomy of learning domains, a widely accepted framework in educational research, categorizes cognitive skills into Lower-Order Cognitive Skills (LOCS) and Higher-Order Cognitive Skills (HOCS) [12]. The former encompasses knowledge/remembering and comprehension/understanding, while the latter includes application/applying, analysis/analyzing, synthesis/creating, and evaluation/evaluating, with application-level questions bridging the gap between the two [13]. Recent research has focused on establishing the connection between cognitive performance and academic achievement [14]. The effective learning process facilitates learners’ understanding and promotes cognitive abilities such as quick thinking (processing speed), information retention (working memory), adaptable goal-oriented responses (cognitive control), and problem-solving skills (reasoning) [15–18]. Consequently, assessing cognitive skills is a better measure for evaluating the effectiveness of different teaching models. To evaluate different cognitive skills, various question formats, such as multiple-choice questions (MCQs), short-answer questions (SAQs), and case study questions (CSQs), are utilized in exams [19]. MCQs are effective in assessing LOCS, including knowledge and comprehension, while SAQs and CSQs are more suitable for evaluating analysis, synthesis, and evaluation of knowledge, serving as a key measure of students’ creative abilities [20]. Thus, evaluating students’ performance across different question types is feasible and valuable for assessing their cognitive skill development in the context of teaching.

Physiology, as the science of life, aims to elucidate the functioning mechanisms of a healthy body. It is a logic-driven course that focuses on fostering clinical reasoning skills in medical students and serves as a prerequisite for all health-related programs [6]. Extensive medical practice has demonstrated that an understanding of physiological mechanisms enables the identification of underlying problems in patients by pinpointing abnormalities in the internal machinery responsible for observable signs and symptoms [21]. Studying physiology trains students’ logical, analytical and knowledge application skills [22]. In light of this, the present study aims to investigate the influence of online and offline teaching models of physiology on the development of cognitive skills in clinical medicine students. By examining the scores of different question types in the final physiology exam, we compared the performance of students enrolled in 2018, who received online instruction, with that of students enrolled in 2019, who experienced offline instruction. This comparison enabled us to assess the impact of different instructional models on students’ cognitive abilities. Furthermore, irrespective of the model of instruction (online or offline), National Quality Open Courses (NQROC) were offered, and student participation contributed to their final grades. Therefore, we analyzed NQROC data from various models of instruction to evaluate student engagement. Our study will contribute to a comprehensive understanding of the effects of online and offline teaching models on the development of cognitive skills in medical education, with significant implications for curriculum design and instructional practices.

## Methods

### Research objects

This study selected 127 students enrolled in clinical medicine in 2018 who undertook online physiology courses in the first semester of 2020, as a consequence of the COVID-19 pandemic. Additionally, 400 students enrolled in 2019 who received face-to-face offline instruction in the first semester of 2021 were included to investigate the comparative impact of online and offline teaching models on the development of cognitive skills. Both groups followed identical course requirements and content. Two groups consisting of 118 and 351 participants, respectively, responded to this study, and all participants provided informed consent. The Research Office of Medical Teaching and Teaching Research at Xiangya School of Medicine, Central South University of China, the authorized body responsible for overseeing undergraduate education and teaching research of medical students, granted approval for our research.

### Teaching implementation

Online teaching model: In 2020, students enrolled in 2018 received physiology instruction through synchronous live lectures utilizing Tencent Meeting (Tencent, Shenzhen, China), an audio and video conferencing software. Prior to each class, the teacher shared the course schedule and learning objectives with students via Tencent QQ (Tencent, Shenzhen, China). During the live online lectures, teachers delivered instruction in accordance with established requirements and objectives, while engaging in online interactions with students. Following the lectures, online tests were administered to assess students’ comprehension of the material.

Offline teaching model: In 2021, students enrolled in 2019 received instruction through traditional face-to-face lectures. Before each class, teachers provided students with the learning objectives for the session. During the face-to-face lectures, teachers adhered to the predetermined requirements and objectives and covered the same content as the online teaching. After class, online tests were administered to assess students’ understanding of the material. The offline group studied the same learning material as the online group.

Both teaching models incorporated the National Quality Open Course (NQROC), which focuses on the fundamental concepts and principles of physiology. The NQROC consists of 118 videos encompassing 11 topics, with the initial ten corresponding to respective chapters, totaling 1080 min, necessitating a minimum viewing time of 900 min by students; The final topic consists of two parts, which are to deepen learners’ science literacy and strengthen academic integrity. The former includes the themes related to the stories of the Nobel Prize Winners in Physiology or Medicine or their novel findings. The latter covers the topics related to the ethical principles of animal experiments and human experiments. These videos were served as supporting materials to broaden learners’ vision and internalize the concept in a casual environment without mandatory requirements (Table 1). Further details on each topic were provided in the supplementary materials. Students were required to watch a minimum of 900 min of video content in order to receive a 20% process grade. The content and requirements of the NQROC were identical for both the online and offline instruction.

**Table 1:** The topics and distribution of NQROC.

Topic	Number of videos
Introduction to Physiology	3
Membrane Physiology and Muscle	14
Blood Cells and blood clotting	10
Cardiovascular Physiology	17
Respiratory Physiology	11
Gastrointestinal Physiology	6
Metabolism and Temperature Regulation	3
Urine formation by the Kidneys	11
Central & Peripheral Neurophysiology	22
Endocrine & Reproductive Physiology	12
Teaching Expansion	9
Total	118

Both teaching models in this study were facilitated by the same team of teachers, consisting of six individuals (two professors and four associate professors). The teaching content was entirely consistent, and all teachers delivered instruction in accordance with the established requirements and objectives prior to each class. Except for the initiator of this study (ZQ Luo, MJ), none of the teachers were aware of the study during the course development, and the analysts were not involved in the instructional implementation.

### Research data

The course examination scores were acquired from the university undergraduate grade management platform. The physiological exam questions employed in this study were sourced from a pre-designed question bank and maintained consistent difficulty levels. Both instructional models adhered to identical examination criteria, encompassing question types and sizes. The examination consisted of a combination of objective and subjective questions, including 50 multiple-choice questions (MCQs) worth 50 points, 8 short-answer questions (SAQs) worth 24 points, and 3 case study questions (CSQs) worth 26 points. The MCQs were automatically scored by the online marking system, while the subjective questions (SAQs and CSQs) were assessed anonymously by two distinct teachers utilizing the online marking system. To mitigate potential subjective biases, the identities of the students and the second teacher were concealed during the scoring process, and the final score for each subjective question was determined as the average of the two independent scores.

The study adhered to the quality evaluation requirements for undergraduate exam papers, including a difficulty coefficient ranging from 0.60 to 0.80, a differentiation greater than 0.20, and a reliability surpassing 0.70 (Table 2). Results indicated that the test papers exhibited moderate difficulty and good differentiation.

**Table 2:** The difficulty coefficient, differentiation, and reliability of the test papers

	Online teaching			Offline teaching		
	all questions	MCQs	SAQs&CSQs	all questions	MCQs	SAQs&CSQs
difficulty coefficient	0.63	0.70	0.56	0.58	0.62	0.54
differentiation	0.38	0.33	0.43	0.41	0.34	0.48
reliability	0.88	0.83	0.75	0.87	0.83	0.73

### Research methods

To assess the learning effectiveness of online and offline teaching models across various score segments, this study employed the methodology proposed by Kelley et al. [23], which highlights the significance of the 27th and 73rd quantiles within a given score distribution. Specifically, high-achieving students were defined as those who scored in the top 27% of the physiology final exam scores (32 students in online teaching and 95 students in offline teaching), while low-achieving students were identified as those who obtained scores in the bottom 27% (32 students in online teaching and 95 students in offline teaching).

The NQROC was incorporated into both online and offline instruction, featuring identical requirements and content, thereby serving as a gauge of students’ learning engagement. Data pertaining to NQROC were sourced from the National Quality Open Course Platform [24], and a standardized value was employed in this study, calculated by dividing a student’s participation by the task completion requirement (Standardized Value = Student’s Participation / Task Completion Requirement). The standardized value for the number of learning videos watched by a student was determined by dividing the actual number of viewed videos by the total number of videos (118). Similarly, the standardized value for the duration of video viewing was derived by dividing the actual viewing time by the task completion requirement of 900 min.

### Statistical analysis

The data were coded, entered, and analyzed using the SPSS statistical package, version 25.0 (SPSS Inc., Chicago, IL). The total score of physiology final exam, scores for each question type, and NQROC learning engagement in both online and offline teaching models exhibited deviations from the normal distribution. Consequently, non-parametric statistical methods were employed, and the results were presented using the median as a measure of central tendency and the upper and lower quartiles to illustrate the discrete trend, denoted as *M* (*P*25, *P*75). To examine the association between the score of the physiology final exam and students’ NQROC learning engagement, Spearman’s rank correlation was utilized. *P* < 0.05 indicated that the difference was statistically significant.

## Results

### Demographics

The cohort comprised 118 students enrolled in 2018 and the remaining students in 2019. The former group underwent physiology instruction during the first semester of 2020, while the latter received instruction during the corresponding period in 2021. All the students were in the clinical medicine major. There were no statistically significant differences both in age and sex between the two groups (Table 3).

**Table 3:** Demographic characteristics of participants

Demographic Characteristics		Online (n = 118)	Offline (n = 351)	*P*
Sex n (%)	Male	51 (43.22)	148 (42.17)	0.802
	Female	67 (56.78)	203 (57.83)	
Age (years)		20.2 ± 0.6	19.9 ± 0.6	0.058

### Academic background of students before studying physiology

To assess the comparability of the two student groups, the Mann-Whitney U test was conducted to compare the scores of courses before studying Physiology - Histology & Embryology, Systematic Anatomy and Biochemistry. The differences were not statistically significant (Table 4). These findings indicated a lack of distinction in the levels of foundational knowledge and learning aptitude between the two cohorts prior to undertaking the physiology course. Consequently, it can be concluded that the academic performance of the two groups of students was comparable.

**Table 4:** Students’ academic background in the two teaching models

Prerequisite Courses	Online *M* (*P*25, *P*75)	Offline *M* (*P*25, *P*75)	*Z*	*P*
Histology & Embryology	68.0 (58.0,75.0)	70.0 (56.5,80.5)	-1.428	0.153
Systematic Anatomy	62.0 (53.0,72.5)	61.0 (55.0,67.0)	-0.872	0.383
Biochemistry	70.0 (58.5,80.0)	68.0 (54.0,78.0)	-0.944	0.345

### Comparison of physiology final exam scores in two teaching models

There was no statistically significant difference in the score of SAQs, CSQs, and total scores in both online and offline teaching models, while the scores of MCQs were higher for all students in online teaching than in offline teaching (Table 5).

**Table 5:** Comparison of the performance of all students in the two teaching models

		Online *M* (*P*25, *P*75)	Offline *M* (*P*25, *P*75)	*Z*	*P*
All students	total score	65.25 (54.75,76.25)	63.50 (52.00,73.00)	-1.503	0.133
	MCQs	36.00 (30.00,43.00)	33.00 (28.00,38.00)	-4.249	< 0.001
	SAQs	12.50 (9.38,18.00)	13.50 (10.00,17.50)	-1.054	0.292
	CSQs	16.00(13.38,18.13)	15.50 (11.50,19.00)	-1.119	0.263

High-achieving students in the online teaching had higher total score of physiology final exam and MCQs than those in the offline teaching, but lower CSQs scores than offline, and there was no statistically significant difference in SAQs (Table 6).

**Table 6:** Comparison of the performance of high-achieving students in the two teaching models

		Online *M* (*P*25, *P*75)	Offline *M* (*P*25, *P*75)	*Z*	*P*
High-achieving students	total score	81.50 (78.50,84.88)	77.75 (75.00,82.88)	-3.001	0.003
	MCQs	44.00 (42.00,46.00)	41.00 (39.00,42.00)	-5.706	< 0.001
	SAQs	19.50 (18.50,20.50)	20.00(18.50,21.50)	-1.588	0.112
	CSQs	20.00(19.00,21.50)	20.50 (20.00,22.50)	-2.775	0.006

The total score, MCQs and CSQs of low-achieving students in the online teaching model were higher than offline, with no statistically significant differences in SAQs (Table 7).

**Table 7:** Comparison of the performance of low-achieving students in the two teaching models

		Online *M* (*P*25, *P*75)	Offline *M* (*P*25, *P*75)	*Z*	*P*
Low-achieving students	total score	50.50 (46.00,52.00)	43.50 (38.50,49.00)	-3.223	0.001
	MCQs	28.00(26.00,29.75)	24.00 (20.00,26.00)	-6.263	< 0.001
	SAQs	6.75 (5.13,8.50)	7.50 (5.50,9.00)	-5.800	0.562
	CSQs	12.00(10.63,12.88)	7.50 (5.00,9.00)	-6.877	< 0.001

### Student’s NQROC learning engagement in two teaching models

Compared to the offline teaching model, both all students and each segment spent in watching videos longer in online teaching model. More videos were watched by all and low-achieving students in online, while the difference in the number of videos watched by high-achieving students was not statistically significant in two teaching models (Table 8).

**Table 8:** Comparison of the number and duration of videos watched by students in the two teaching models

NQROC Learning Engagement		Online *M* (*P*25, *P*75)	Offline *M* (*P*25, *P*75)	*Z*	*P*
Duration (min)	All students	1224(711,1611)	558 (72, 783)	-10.350	< 0.001
	High-achieving	1341 (909,1512)	621 (108, 864)	-5.776	< 0.001
	Low-achieving	891 (396,1530)	468 (27, 801)	-3.705	< 0.001
Number	All students	118(78,118)	98(40,114)	-6.826	< 0.001
	High-achieving	118(105,118)	106(44,114)	-5.077	0.530
	Low-achieving	109(48,118)	85(14,116)	-2.351	0.019

### Correlation between students’ total score and NQROC learning engagement

In online teaching model, a statistically significant positive correlation was found between students’ total scores of physiology final exam, MCQs and NQROC learning engagement, with *r*_*s*_ of 0.227 (*P* = 0.002) and 0.192 (*P* = 0.037), respectively. However, no significant correlation was observed between SAQs, CSQs and engagement (*P* > 0.05). There was a positive correlation between students’ total score of physiology final exam, SAQs and NQROC learning engagement in offline teaching with *r*_*s*_ of 0.113 (*P* = 0.034) and 0.142 (*P* = 0.008) respectively, while there was no significant correlation between MCQs, CSQs and engagement (*P* > 0.05).

## Discussion

The present study sought to investigate the impact of online and offline teaching models on the cognitive skills of students across different score segments. This research made a significant contribution to the existing literature by elucidating the varying effects of these two distinct teaching models (online and offline) on the enhancement of cognitive skills. The results of this study indicated that there was no significant difference in academic performance between the two teaching models across all score segments. However, students who received online instruction performed better on MCQs. These findings supported previous research suggesting that students generally hold a positive attitude towards online teaching [25, 26]. Moreover, previous studies have demonstrated that online learning can enhance the knowledge and skills of medical students [27]. Notably, MCQs primarily assess lower-order cognitive skills [20], involving the retrieval of factual knowledge [13]. Our study suggested that online teaching had the potential to improve these skills, as evidenced by the superior performance of students who received online instruction on MCQs. One possible explanation for this finding was that in the online teaching model, students encountered more challenges in seeking immediate assistance from teachers, leading them to rely on online resources such as NQROC. Consequently, students fully utilized and repeatedly engaged with these resources, focusing on fundamental concepts that were essential elements of MCQs. This heightened engagement with online materials may result in improved performance in memory-related areas, as reflected by the statistics showing that students using the online teaching model spend more time watching videos. However, some research suggested that students may be less active in online courses compared to offline [8]. This implied that students’ retention of knowledge from in-class sessions may be affected, requiring additional time for reviewing and reinforcing their memory compared to offline teaching. Overall, our findings confirmed that online teaching had its own characteristics that were more conducive to LOCS than offline teaching, but not to the development of HOCS. Also, in motor and attitude domains, it was discovered that it may not be as effective as traditional teaching, as it may reduce student concentration in the course [28].

Furthermore, the results revealed that both low-achieving and high-achieving students in online teaching performed better on the total score and MCQs. However, high-achieving students in online teaching scored lower on CSQs, whereas low-achieving students outperformed those in offline teaching. CSQs primarily assess higher-order cognitive skills such as analysis, evaluation and creation, which require students to synthesize and apply knowledge based on memory and understanding [29, 30]. This discrepancy could be attributed to the limited depth of teaching when adopting online teaching, the lack of effective communication between teachers and students, and the initial challenges in adapting to live online teaching of the teachers and students for the first time, which may impede the development of higher-order cognitive skills in high-achieving students compared to offline teaching. In contrast, offline teaching allowed for more comprehensive communication between students and teachers. The teacher can adjust the teaching progress based on timely on-site feedback from students, facilitating the expansion of teaching and enhancing the development of students’ logical and analytical skills, particularly among high-achieving students who possess self-discipline and better interact with the teacher. Therefore, in the online teaching model, it is crucial for teachers to strengthen the construction of online learning forums to meet the learning depth requirements of high-achieving students [31]. The better performance of low-achieving students on CSQs in the online model may be attributed to their increased time spent watching videos compared to offline teaching. This increased utilization of NQROC resources in online learning is more conducive to the mastery of knowledge by low-achieving students.

Moreover, the study revealed that students exhibited greater NQROC learning engagement in online teaching, as evidenced by positive correlations between total scores and NQROC engagement, which were stronger compared to offline teaching. This finding aligned with previous research on self-regulated learning behavior [1], suggesting that self-regulation fostered students’ confidence in their abilities and enhanced motivation for self-learning [32], thereby potentially contributing to the effectiveness of online teaching. Additionally, the study found that the duration of video viewing was longer in online teaching, indicating that the comprehensive utilization and repeated viewing of online resources could improve performance in memory-related areas. In the offline teaching model, since teachers already delivered lectures effectively in a face-to-face format in the classroom, high-achieving students possessed a strong grasp of the foundational knowledge. In contrast, the NQROC resources were only available in a 10-15-minute video format, lacking in-depth explanations of knowledge. Therefore, high-achieving students relied less on NQROC resources after receiving offline face-to-face instruction. Furthermore, previous research has indicated that some students prefer longer videos with in-depth explanations of knowledge [33]. Overall, this study provided insights into the potential benefits and drawbacks of different teaching models, as well as their effects on the development of cognitive skills in students. These findings had practical implications for the design and implementation of effective online and offline teaching, particularly for the development of higher-order cognitive skills.

### Limitations

It is worth noting that the findings of this study are specific to the physiology, and courses with distinct characteristics (those focusing on morphology study or body function) may yield different conclusions. In order to comprehensively evaluate the effectiveness of an instructional model, a mixed methods approach incorporating both quantitative and qualitative research methods, such as empirical studies and case interviews, would be more appropriate. By incorporating diverse research methods, richer information can be obtained, including teachers’ feedback and students’ learning experiences, which would enhance the depth and breadth of the study’s findings.

## Conclusion

In conclusion, this study has provided evidence to suggest that online teaching exhibits comparable effectiveness to offline teaching within the cognitive domain. This assertion is corroborated by the observation that the online teaching model facilitates the advancement of students’ lower-order cognitive skills through their enhanced utilization of NQROC resources. Notably, online teaching engenders greater student engagement and manifests more active self-directed learning behavior in comparison to offline teaching. Nonetheless, it is essential to acknowledge that online teaching may not be conducive to the cultivation of higher-order cognitive skills in high-achieving students, primarily due to constraints on teacher-student communication and the limited duration of individual instructional videos. Consequently, within the online teaching model, it is imperative for educators to prioritize the reinforcement of teacher-student interactions, foster active student participation, and facilitate the establishment of online learning forums to augment the depth of learning, particularly among high-achieving students.

### Electronic supplementary material

Below is the link to the electronic supplementary material.

Supplementary Material 1

## Data Availability

All data generated or analysed during this study are included in this published article.

## Abbreviations

NQROC: National quality open courses
SAQs: short-answer questions
CSQs: case study questions
MCQs: multiple-choice questions
LOCS: lower-order cognitive skills
HOCS: higher-order cognitive skills
