# Supplementary material for: Exploring the impact of online and offline teaching methods on the cognitive abilities of medical students: a comparative study
Source: BMC Med Educ. 2023 Aug 8;23:557. doi: 10.1186/s12909-023-04549-x (PMC10410817; doi:10.1186/s12909-023-04549-x)
Supplement: Supplementary file 1 — Supplementary Material 1 [file 12909_2023_4549_MOESM1_ESM.docx]

**sTable 1** The core concepts and principles of topics of the National quality open courses (NQROC).

| **Topic** | **Number of videos** | **Content of videos** |
| --- | --- | --- |
| Introduction to Physiology | 3 | 1. The Guidance of Medical Physiology |
|  |  | 1. Homeostasis |
|  |  | 1. Regulation of body functions |
| Membrane Physiology and Muscle | 14 | 1. The Guidance of Cell |
|  |  | 1. Transport of Substances Through Cell Membranes |
|  |  | 1. Resting Potential |
|  |  | 1. Action Potential (Ⅰ) |
|  |  | 1. Action Potential (Ⅱ) |
|  |  | 1. Forces acting across the cell membrane on ions |
|  |  | 1. Initiation of the action potential |
|  |  | 1. Electrotonic potential & Acute subthreshold potential |
|  |  | 1. Propagation of the action potential |
|  |  | 1. Refractory period after an action potential |
|  |  | 1. Neuromuscular Transmission |
|  |  | 1. Molecular mechanism of muscle contraction |
|  |  | 1. Excitation-contraction Coupling |
|  |  | 1. Characteristics of whole muscle contraction |
| Blood Cells and blood clotting | 10 | 1. The Guidance of Blood |
|  |  | 1. Plasma osmotic pressure |
|  |  | 1. Physiological characteristics of red blood cells |
|  |  | 1. Production and Destruction of Red Blood Cells |
|  |  | 1. Physical and Chemical Characteristics of Platelets |
|  |  | 1. Functional characteristics of Platelets |
|  |  | 1. Blood Coagulation |
|  |  | 1. Fibrinolysis |
|  |  | 1. O-A-B Blood Type System |
|  |  | 1. Rh Blood Type System |
| Cardiovascular Physiology | 17 | 1. The Guidance of Circulation |
|  |  | 2. The Cardiac Cycle and Heart Pumping |
|  |  | 3. Regulation of heart pumping - intrinsic cardiac pumping regulation |
|  |  | 1. Effect of Arterial Blood Pressure load on Cardiac Output |
|  |  | 1. Normal Values for Cardiac Output |
|  |  | 1. Monophasic action potential of ventricular muscle fiber |
|  |  | 1. Monophasic action potential of Purkinje Cells and Sinus Node Cells |
|  |  | 1. Waveforms of the Normal Electrocardiogram |
|  |  | 1. Excitability of Cardiomyocytes |
|  |  | 1. Conductivity of Cardiomyocytes |
|  |  | 1. Contractility of Cardiomyocytes |
|  |  | 1. The Formation of Arterial Blood Pressure and Its Influencing Factors |
|  |  | 1. Venous Blood Pressure and Venous Return |
|  |  | 1. Microcirculation and Equilibration with interstitial fluid |
|  |  | 1. Neural Control of Cardiovascular System |
|  |  | 1. Humoral Control of Cardiovascular System |
|  |  | 1. Coronary Circulation |
| Respiratory Physiology | 11 | 1. The Guidance of Respiration |
|  |  | 1. Mechanics of Pulmonary Ventilation |
|  |  | 1. Effect of the Thoracic Cage on Lung Expansibility |
|  |  | 1. Surfactant, Surface Tension, and Collapse of the Alveoli |
|  |  | 1. Inelastic Resistance to Pulmonary Ventilation |
|  |  | 1. Pulmonary Volumes and Capacities |
|  |  | 1. Gas Exchange in the Lungs |
|  |  | 1. Oxygen Transport |
|  |  | 1. Carbon Dioxide Transport |
|  |  | 1. Chemical control of respiration |
|  |  | 1. The Hering-Breuer inflation reflex |
| Gastrointestinal Physiology | 6 | 1. The Guidance of Digestion and Absorption |
|  |  | 1. Gastric Secretion |
|  |  | 1. Regulation of Gastric Secretion |
|  |  | 1. The Motor Functions of the Stomach |
|  |  | 1. Pancreatic and Biliary Secretion |
|  |  | 1. Absorption in the Small Intestine |
| Metabolism and Temperature Regulation | 3 | 1. The Guidance of Energy Metabolism and Temperature Regulation |
|  |  | 1. Energy Metabolism—Factors That Influence Energy Output |
|  |  | 1. Body Temperature and Regulation |
| Urine formation by the Kidneys | 11 | 1. The Guidance of the Urinary System |
|  |  | 1. Glomerular Filtration |
|  |  | 1. Proximal Tubular Reabsorption |
|  |  | 1. The Glucose Transport System in the Proximal Tubule |
|  |  | 1. Loop of Henle, Distal Tubule and Collecting Ducts Reabsorption |
|  |  | 1. Secretion Along Different Parts of the Nephron |
|  |  | 1. Establishment and Maintenance of Renal Medullary Hypertonicity |
|  |  | 1. Excreting a Concentrated/ Dilute Urine |
|  |  | 1. Regulation of the Glomerular Filtration Rate |
|  |  | 1. Regulation of Urine Formation - Autoregulation and Neuroregulation |
|  |  | 11. Regulation of Urine Formation - Humoral Regulation |
| Central & Peripheral Neurophysiology | 22 | 1. The Guidance of the Sensory Organs |
|  |  | 2. Overview of the Sensory Organs |
|  |  | 3. Optics of Vision |
|  |  | 4. Receptor and Neural Function of the Retina |
|  |  | 5. Conduction of sound from the Tympanic Membrane to the Cochlea |
|  |  | 6. Function of the Cochlea and the Organ of Corti |
|  |  | 7. The Guidance of Nervous system |
|  |  | 8. Synaptic Transmission |
|  |  | 9. Neurotransmitters and Corresponding Receptors |
|  |  | 10. Several Important Neurotransmitters and Corresponding Receptors |
|  |  | 11. General Properties of Reflexes |
|  |  | 12. Central Integration of Reflexes |
|  |  | 13. Sensory function of the Nervous System |
|  |  | 14. Pain |
|  |  | 15. Stretch Reflex (Ⅰ) |
|  |  | 16. Stretch Reflex (Ⅱ) |
|  |  | 17. Plan, Sequence, and Time Complex Movements |
|  |  | 18. Function of the Basal Ganglia in Executing Patterns of Motor Activity |
|  |  | 1. Function of the Cerebellum in Overall Motor Control |
|  |  | 20. The Autonomic nervous system |
|  |  | 21. Brain Electrical Activity and Wakefulness and Sleep |
|  |  | 22. Learning and Memory |
| Endocrine & Reproductive Physiology | 12 | 1. The Guidance of Endocrinology |
|  |  | 2. Physiological Function of Growth Hormone |
|  |  | 3. Endocrine of the Thyroid Gland (Ⅰ) |
|  |  | 4. Endocrine of the Thyroid Gland (Ⅱ) |
|  |  | 5. Adrenocortical Hormones (Ⅰ) |
|  |  | 1. Adrenocortical Hormones (Ⅱ) |
|  |  | 7. Insulin |
|  |  | 8. The Guidance of Reproduction |
|  |  | 9. Endocrine of the Ovary and Function of female Hormones |
|  |  | 10. Monthly Endometrial Cycle and Menstruation |
|  |  | 11. Regulation of female Monthly Rhythm |
|  |  | 12. Endocrine of the Testes and Function of male Hormones |
| Supporting materials for deepening science training | 9 | 1. Homeostasis & Systems Medicine |
|  |  | 1. The History of Blood Transfusions - The Discovery of O-A-B Blood Type System |
|  |  | 1. A Fantastic Dream and Otto Loewe's Nobel Prize |
|  |  | 1. The Paradox of Acetylcholine and Nobel's Confusion - The Discovery of Nitric Oxide |
|  |  | 1. The Discovery of Helicobacter pylori |
|  |  | 1. The Father of Insulin - Youngest Nobel Laureate |
|  |  | 1. Pavlov's Regret - The Discovery of The First Hormone |
|  |  | 8. Ethical Principles for Animal Experimentation |
|  |  | 9. Ethical Principles of Human Experimentation |
| Total | 118 |  |
